# Integrating the PD-L1 Prognostic Biomarker in Non-Muscle Invasive Bladder Cancer in Clinical Practice—A Comprehensive Review on State-of-the-Art Advances and Critical Issues

**DOI:** 10.3390/jcm13082182

**Published:** 2024-04-10

**Authors:** Francesca Sanguedolce, Ugo Giovanni Falagario, Magda Zanelli, Andrea Palicelli, Maurizio Zizzo, Gian Maria Busetto, Angelo Cormio, Giuseppe Carrieri, Luigi Cormio

**Affiliations:** 1 Pathology Unit, Policlinico Foggia, University of Foggia, 71122 Foggia, Italy; francesca.sanguedolce@unifg.it; 2 Department of Urology and Renal Transplantation, Policlinico Foggia, University of Foggia, 71122 Foggia, Italy; ugo.falagario@unifg.it (U.G.F.); gianmaria.busetto@unifg.it (G.M.B.); giuseppe.carrieri@unifg.it (G.C.); luigi.cormio@unifg.it (L.C.); 3 Pathology Unit, Azienda USL-IRCCS di Reggio Emilia, 42123 Reggio Emilia, Italy; magda.zanelli@ausl.re.it (M.Z.); andreapalicelli@hotmail.it (A.P.); 4 Surgical Oncology Unit, Azienda USL-IRCCS di Reggio Emilia, 42123 Reggio Emilia, Italy; maurizio.zizzo@ausl.re.it; 5 Urology Unit, Azienda Ospedaliero-Universitaria Ospedali Riuniti Di Ancona, Università Politecnica Delle Marche, Via Conca 71, 60126 Ancona, Italy; 6 Department of Urology, Bonomo Teaching Hospital, 76123 Andria, Italy

**Keywords:** PD-L1, bladder cancer, NMIBC, biomarker

## Abstract

Bladder cancer (BC) is one of the most prevalent cancers worldwide. Non-muscle invasive bladder cancer (NMIBC), comprising the majority of initial BC presentations, requires accurate risk stratification for optimal management. This review explores the evolving role of programmed cell death ligand 1 (PD-L1) as a prognostic biomarker in NMIBC, with a particular focus on its implications in the context of Bacillus Calmette-Guérin (BCG) immunotherapy. The literature suggests a potential association between elevated PD-L1 status and adverse outcomes, resistance to BCG treatment, and disease progression. However, conflicting findings and methodological issues highlight the heterogeneity of PD-L1 assessment in NMIBC, probably due to the complex biological mechanisms that regulate the interaction between PD-L1 and the tumor microenvironment. The identification of PD-L1 as a prognostic biomarker provides ground for tailored therapeutic interventions, including immune checkpoint inhibitors (ICIs). Nevertheless, challenges such as intratumoral heterogeneity and technical issues underscore the need for standardized protocols and larger, homogeneous trials. This review contributes to the ongoing debate on the personalized management of NMIBC patients, focusing on the advances and perspectives of incorporating PD-L1 as a biomarker in this setting.

## 1. Introduction

### 1.1. Clinical Aspects of Non-Muscle Invasive Bladder Cancer (NMIBC)

Bladder cancer (BC) poses a significant global health burden, ranking seventh among the most prevalent cancers in both genders worldwide in 2022 and raising to the fourth position when considering only males [1]. Approximately 75% of BC patients initially present with non-muscle-invasive mucosa (stage Ta, CIS)- or submucosa (stage T1)-confined disease (NMIBC) [2], especially younger patients [3]. The standard of care for high-risk NMIBC spans from transurethral resection (TUR), with the aim to remove visible lesions and provide specimens for pathological examination, and intravesical instillation (mostly using Bacillus Calmette-Guérin (BCG)), to radical cystectomy in high-risk cases [4]. Though BCG immunotherapy stands as the main treatment for this subset of tumors, approximately 30% of patients are non-responders, possibly due to several clinical and pathological factors, including multifocality, lymphovascular invasion, and a high grade on the re-transurethral resection of the bladder (re-TURB) [5].

Furthermore, BCG treatment may be challenging in that local and systemic side effects have been reported, which may be severe in <5% of patients [4]. Such effects, along with BCG infections after instillations and interruptions in BCG availability, may result in treatment discontinuation [6], and even in the absence of such drawbacks, a notable percentage of patients experience treatment failure, recurrence, or progression of the disease, highlighting the need to unravel the underlying mechanisms driving these outcomes in order to identify those BCG non-responders who might benefit of early alternative treatments. The clinical and biological heterogeneity NMIBC makes it essential to search for biomarkers capable to identify risk categories for the proper management of these patients. Several molecules have been examined as prognostic biomarkers in NMIBC over the years, some of which are feasible as therapeutic targets as well, namely cell cycle proteins (p53, pRB), transcription factors (HER2), proliferation marker Ki-67, mismatch repair proteins (hMLH1, hMSH2), cadherins, surviving, and androgen receptors [7,8]. The availability of tissue samples used for diagnosis makes the possibility of assessing new molecules attractive through immunohistochemical methods, using antibodies that can be easily incorporated into routine practice. However, so far, no tissue biomarker has been included into risk categorization tools. Therefore, the search for reliable prognostic biomarkers is still ongoing.

### 1.2. PD-L1: Biological Mechanisms

Programmed cell death ligand 1 (PD-L1) is a cell surface glycoprotein and a member of the B7/CD28 co-stimulatory factor superfamily [9]; it is expressed on different types of immune cells (IC-PD-L1) and tumor cells (TC-PD-L1) [10,11]. As an inhibitor of the host immune response, PD-L1 induces apoptosis and/or inhibition of tumor-specific T cell activation and proliferation, cytolytic function, and cytokine production, mostly by binding to programmed cell death-1 (PD-1) receptor [12]. Similarly, cancer cells can escape immune surveillance by upregulating PD-L1, resulting in tumor growth [11]. Both TC-PD-L1 and IC-PD-L1 expression can occur in cancer due to persistent antigenic stimulation, with subsequent cytokine expression upon T cell activation, which, in turn, can induce PD-L1 on surrounding ICs and TCs [13]. Moreover, the PD-1/PD-L1 signaling axis may induce immune inhibitory/exhaustion signaling of activated T cells and thus significantly impair the anti-tumor immune response [14]. PD-L1 can also interact with other molecules and pathways (IL-6/STAT3, EMT) involved in tumor development and aggressiveness [15]. Furthermore, several pathways have been suggested to be involved in the process of bladder carcinogenesis associated with PD-L1 induction, such as the PI3K/AKT/PTEN and JAK/STAT pathways [16,17].

### 1.3. PD-L1 in Bladder Cancer: Clinical Implications 

Since a blockade of the PD-1/PD-L1 signaling pathway may restore the native T-cell mediated tumor response, leading to tumor regression [18], in the last few years, an increasing number of immune checkpoint inhibitors (ICI) have been developed and applied in the management of different types of cancers [19]. Currently, ICI (including PD-1 and PD-L1 inhibitors) are administered either as maintenance therapy for patients treated with first-line platinum-based chemotherapy who do not experience disease progression, or as first-line regimen for those patients ineligible to receive cisplatin [20,21]. According to a timely systematic review and meta-analysis on the efficacy of ICI in MIBC [22], PD-L1 inhibitors are less effective alone than in combination with other ICI or chemotherapeutic agents in terms of lower, complete, or partial, pathological response, yet they are associated with severe immune-related adverse effects. As BC patients treated with PD-L1 inhibitors show favorable OS and RFS, this treatment seems to be a promising therapeutic option for selected MIBC patients, highlighting the need to find feasible biomarkers.

### 1.4. Prognostic Role of PD-L1 in BC: The Big Picture 

Evidence from the literature has shown that pretreatment PD-L1 overexpression is associated with poor outcome in terms of shorter prognosis and resistance to immune therapies in multiple cancers, including colorectal, lung, kidney, head and neck, pancreatic, and gastric cancer [23,24,25,26,27,28,29,30]. Several studies have shown a correlation between PD-L1 expression in BC samples and multiple clinical and pathological parameters of poor prognosis, including high tumor grade, increased resistance to BCG therapy, and muscle-invasive disease [31,32], although there are discrepancies in the literature on this topic [33]. So far, conflicting findings have been reported regarding the prognostic role of PD-L1 expression in BC. While some authors have revealed worse survival rates in BC patients with elevated PD-L1 expression [34,35,36,37,38], others have failed to find such a correlation [39,40]. The prognostic role of PD-L1 has recently been the subject of five meta-analyses over a few recent years (2019–2022), with a variable number of studies [11,12,13,14,15,16,17,18,19,20,21,22,23,24,25,26,27] and cases (1393–4032) assessed in each analysis due to different eligibility criteria and search strategy variables (including databases, interval time, and MeSH terms) [41,42,43,44,45] (Supplementary Table S1). The authors found a statistically significant association overall between the expression of TC PD-L1 and poorer outcome parameters. Furthermore, in three papers [41,42,43], a significant direct correlation with disease stage has been reported. Obviously, meta-analyses have well-known limitations, partly intrinsic to the methodology itself, mainly (1) heterogeneity in search criteria and data collection; (2) variations in clinical and pathological characteristics, as well as treatments among the examined populations; (3) small sample sizes in some studies, resulting in lack of power for the analysis; and (4) publication bias caused by the trend of publishing positive results more easily than negative ones. Interestingly, Ding et al. [42] observed that the meta-analysis of 11 studies on disease stage showed a significantly higher incidence of TC PD-L1 expression in MIBCs as compared to NMIBC (OR = 3.67, 95% CI: 2.53–5.33), whereas no correlation was found when assessing IC PD-L1. Furthermore, a consistent inverse association between TC PD-L1 expression and treatment with BCG before cystectomy was reported (OR = 0.39, 95% CI: 0.18–0.82). So far, no review exists on the prognostic value of PD-L1 in NMIBC. Therefore, in this study, we assessed the available evidence to explore the expression of PD-L1 in association with clinicopathological factors and outcomes of NMIBC patients and provided a detailed discussion on issues and unmet needs.

## 2. Materials and Methods

**Figure 1:**
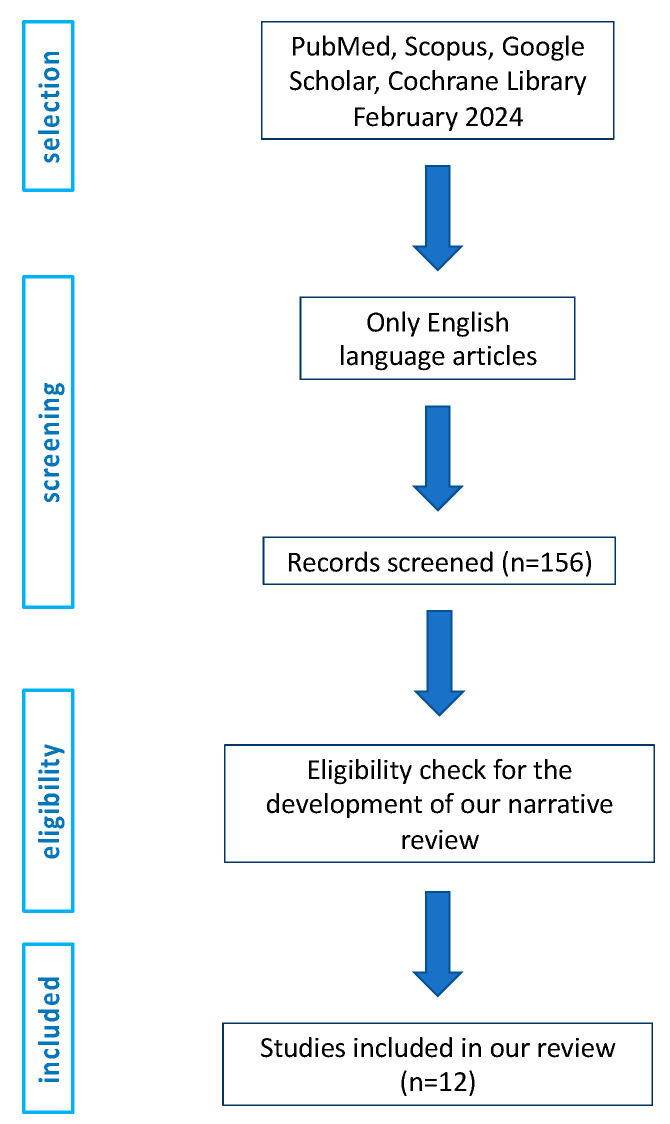
Flow chart.

A thorough search of literature databases (PubMed, Scopus, Google Scholar, and the Cochrane Library) was conducted in February 2024. The search used carefully selected terms that matched the focus of our work (“PD-L1”, “non muscle invasive bladder cancer”, and “NMIBC”). Only peer-reviewed articles in English were included. In the early phase, two authors meticulously screened titles as well as abstracts to identify and select manuscripts that met the criteria for inclusion, namely, original articles assessing the prognostic role of PD-L1 in cohorts of NMIBC. We screened 156 articles (title/abstract). The flow chart is presented in Figure 1.

## 3. Results and Discussion

### 3.1. Prognostic Role of PD-L1 in NMIBC: Main Findings

The main findings from selected studies aiming to explore the prognostic role of PD-L1 in NMIBC are summarized in Table 1. Semeniuk-Wojtaś et al. reported a significant association between ≥1% TC PD-L1 and an unfavorable outcome due to a higher probability of cancer recurrence after the first year since the first TURB [46]. Accordingly, Romiguié et al. [47] found that TC PD-L1 expression, assessed as a continuous variable, was an independent prognostic factor for disease-free survival (DFS). Conversely, according to Eich et al., tumors showing higher TC PD-L1 expression (at both 1% and 5% cut-offs) had a lower disease grade and recurrence risk at any following biopsy or cystoscopy [48]. In keeping with this, Eckstein et al. [49] reported a better overall survival in a cohort of MIBCs tumors with >1% IC-PD-L1 expression. Other authors have failed to find a prognostic significance of PD-L1 status in NMIBC [50,51], in keeping with previous findings on MIBC studies [40]. The higher recurrence risk and/or shorter recurrence-free survival (RFS) observed in NMIBC patients who show an increased TC PD-L1 expression, as reported by several studies [46,47,52], has a significant clinical implication, in that it prompts the need to enhance the follow-up of these patients. In this setting, a closer monitoring of the disease for a few years after the first diagnosis of NMIBC, through a protocol that includes more frequent cystoscopies with a shorter interval between them, would be advisable [46]. The occurrence of a statistically significant difference in PD-L1 expression based on the stage has been observed by some authors [47,48,51,53], possibly due to discrepancies in the tumor microenvironment or genomic drivers between MIBC and NMIBC [54]. For instance, Huang et al. [55] examined a cohort of BCs of different stages, finding that increased levels of PD-L1 mRNA expression in tumors with higher stage and shorter survival. Accordingly, Eich et al. reported that tumors with higher IC PD-L1 expression, as assessed in peritumoral lymphocytes, had a significantly higher (≥pT1) stage, as compared with noninvasive lesions [48], as well as a trend toward an increased risk of tumor stage progression at any biopsy. These findings highlight the need for using different assessment methods in recording PD-L1 in NMIBC and MIBC settings. Roumiguié et al. [47] achieved statistically significant results regarding the prognostic value of PD-L1 for DFS only when assessing its expression as a continuous variable, rather than defining it on the basis of the cut-offs used in trials evaluating treatment response in cohorts of advanced/metastatic BC [56,57]. Therefore, it is not possible to establish a single scoring system for assessing PD-L1 expression in BC. Rather, selection should be based on the methodology and antibodies used, the patients’ cohort, and the purpose of the study (prognostic or predictive).

In conclusion, the analysis of PD-L1 expression in BC reveals divergent prognostic implications depending on tumor stage and assessment methodology. While higher TC PD-L1 expression seems to correlate with poorer outcomes in NMIBC, the significance of PD-L1 status remains inconclusive in some studies. The varying prognostic value underscores the necessity for standardized assessment methods and careful interpretation when evaluating PD-L1 expression in BC.

### 3.2. Prognostic Role of PD-L1 in NMIBC: Focus on BCG Immunotherapy

Currently, the role of PD-L1 as a prognostic factor for BCG response in HR-NMIBC remains unclear. Even studies with a large number of patients provided discrepant findings, suggesting that the assessment of PD-L1 status is not a robust predictor of BCG response [58,59]. A few studies have shown a positive correlation between increased PD-L1 expression and BCG unresponsiveness [60,61], highlighting the dynamicity of PD-L1 depending on the time of analysis and treatment in this setting. 

Kates et al. observed a significantly increased PD-L1 (SP142, 22C3) expression before treatment in the subgroup of 32 BCG non-responders as compared to 31 responders (0–4% vs. 25–28%, *p* < 0.01) in their cohort of HG-NMIBCs, including CIS, Ta, and T1 tumors [60]. Their findings suggest that underlying resistance to BCG might result from pretreatment immune exhaustion and adaptive immune responses, which, in turn, may lead to BCG failure and BC recurrence in up to 25% patients. Pierconti et al. assessed the PD-L1 status in a homogeneous cohort of 60 CIS tumors [61], finding higher TC and IC PD-L1 rates in BCG-non-responders than in BCG responders, though a significant association with recurrence was observed only with TC PD-L1 (22C3) expression (*p* = 0.035). Inman et al. identified PD-L1 staining in approximately 40% of CIS tumors and reported increased rates of PD-L1 expression and/or BCG-induced granuloma occurring close to recurrent tumors after BCG immunotherapy. Furthermore, approximately 15–20-fold more diffuse and intense PD-L1 expression within such BCG-induced granulomata was observed in non-responders after vs. before treatment [31]. The authors hypothesized that an accumulation of PD-L1 in IC granuloma may lead to a decreased interaction of T lymphocytes with antigen-presenting cells, resulting in a lack of BCG response. Accordingly, Hashizume et al. observed a consistent increase in TC and IC PD-L1 expression levels (*p* < 0.001 and *p* = 0.030, respectively) after BCG treatment in BCG-resistant and recurrence-free NMIBC patients [62], possibly due to cancer cells evading immune recognition through PD-L1 upregulation. All in all, these studies seem to imply the role of increased PD-L1 expression in supporting cancer cells to escape from the killing effects of tumor-specific immune cells elicited by BCG treatment. Obviously, such findings provide grounds for the use of PD-L1 antibody immunotherapy and/or ICI-BCG combined regimen, as an effective treatment option after BCG failure, which is the rationale behind ongoing clinical trials [63,64,65]. 

Conversely, in the study by Civriz et al., no association was found between PD-L1 (E1L3N) status, assessed through different scoring systems and cut-offs, and recurrence after BCG treatment [53], in keeping with the findings of previous studies [50,59]. According to Aydin et al. [59], neither pre-treatment nor post-treatment PD-L1 (SP142) expression was associated with RFS or progression-free survival (PFS), even using different cut-offs (≥3% or ≥5%), in a large study cohort of 117 HG-NMIBCs. Furthermore, these authors observed the downregulation of PD-L1 expression in patients with refractory recurrence, possibly due to more complex anti-tumor mechanisms involving the PD-L1 pathway. In a previous study reporting no association between prior adjuvant BCG treatment and TC/IC PD-L1 expression in 69 urothelial carcinomas, detailed clinicopathological information were not provided [40]. Delcourt et al. failed to report an association between PD-L1 (E1N3L) expression and early recurrence in a large cohort of 186 NMIBCs. Nevertheless, only the induction of BCG was administered to the patients in their cohort, resulting in a possible bias in the result interpretation [58]. 

Putative reasons which may contribute to affect the consistency and comparability of available findings in this setting (and more generally the assessment of PD-L1 expression as a biomarker in BC) are (1) intrinsic to the study population, including a small number of patients overall [50,58,62], an exceedingly low recurrence rate after BCG treatment, and/or unbalanced subgroup size (i.e., BCG responders vs. non responders) [53]; (2) intrinsic to the treatment, including the frequency and/or timing of BCG administration and type of BCG reagents; (3) intrinsic to the method, such as PD-L1 assessment through heterogeneous assays, antibody clones, cellular populations, scoring systems, and cut-off points (see next section); and (4) intrinsic to the tumor/tissue sample, due to the intra-tumoral heterogeneity of PD-L1 expression and the dynamic nature of the tumor microenvironment [66,67]. Furthermore, the constantly changing of the molecule over time (temporal heterogeneity) has been proven by the discrepancy over different tumor stages, as well as between metastatic sites and corresponding primary tumors, revealed by several authors [68,69].

Variable and sometimes contradictory findings have been reported regarding the assessment of PD-L1 as a prognostic factor for BCG response in HR-NMIBC, with some studies suggesting a positive correlation between increased PD-L1 expression and BCG unresponsiveness, whereas others have failed to establish a significant predictive association. Factors contributing to such inconsistency are related to heterogeneity of patient cohorts, BCG treatment regimens, and methodologies for PD-L1 assessment. Moreover, the dynamic nature of PD-L1 expression over time and its interaction with the tumor microenvironment play a role in creating a more complex landscape. 

### 3.3. PD-L1 Expression on Immune Cells and Interaction with BCG Immunotherapy

Overall, IC PD-L1 expression rates are higher than their TC counterpart (see Table 1). Delcourt et al. [58] observed a consistent association between IC PD-L1 expression and immune infiltrate density, in keeping with the results by Inman et al. [31] and Breyer et al. [70], with the latter assessing PD-L1 expression through the measurement of its mRNA level by quantitative RT-PCR. Wankowitz et al. [51] reported a similarly wide IC PD-L1 expression in T1 HG tumors as in MIBCs, with the latter showing consistently higher TC PDL1 status (*p* = 0.001). Blinova et al. observed a higher number of PD-L1-expressing CD8+ cells in chemotherapy- and immunotherapy-naive relapsed NMIBC, as well as in luminal and double-negative, high-grade basal relapsed UC after previous frontline BCG treatment [52]. Accordingly, a parallel increase in CD8+ cells and PD-L1 (E1L3N) expression after BCG treatment was reported by Hashizume et al. [62]. In keeping with the adaptive immune resistance and immune escape hypothesis supported by these findings, Kates et al. [60] showed that IC PD-L1 expression was increased in BCG non-responders and co-localized with CD8+ cells, with a higher density of CD8+ cells in PD-L1-positive areas and consistently higher CD8+ cell counts in post-BCG samples from both responders and non-responders (*p* = 0.017). Moreover, there were significantly lower pre-treatment CD4+ cell counts in PD-L1-positive BCG responders as compared to PD-L1-negative samples (12% vs. 50%, *p* < 0.01), suggesting that inefficient CD4+ trafficking, possibly due to hampered cytokine expression/interaction may be another mechanism of BCG resistance. In the process of adaptive immune resistance, the recruitment of cytotoxic CD8+ T cells within tumor tissues resulted in these cells producing IFN-γ in order to elicit a cytotoxic antitumor response. Subsequently, the activation of these signaling pathways upregulates PD-L1 in surrounding TCs and ICs to escape recognition by such cytotoxic immune cells [67]. BCG immunotherapy exerts its anti-tumor activity by accelerating the infiltration of CD8+ cells in the tumor microenvironment [31], as well as by shifting the CD4+ Th2 to Th1 type immune responses [71,72]. Furthermore, it has been shown that BCG infection of urothelial cells can induce PD-L1+ Tregs (a subset of CD4+ cells), partially via an IFN-α-mediated mechanism, and high levels of urinary Tregs are associated with early recurrence after BCG treatment [73], suggesting that the interaction between PD-L1 and the different cell types in the tumor microenvironment is far more complex, especially in the setting of BCG treatment of NMIBC. Interestingly, only one study addressed the association between PD-L1 status and IC levels in peripheral blood, reporting lower peripheral lymphocyte counts and higher neutrophil-to-lymphocyte ratios (NLR) in patients with higher local PD-L1 expression, suggesting a possible relation between local immunosuppression and systemic inflammation, which may be combined as prognostic markers in NMIBC patients [50].

All in all, the discrepancy between IC and TC PD-L1 expression rates highlights the complexity of the immune microenvironment in BC. There is strong evidence supporting higher IC PD-L1 expression in these patients, showing significant association with immune infiltrate density and CD8+ cell localization, particularly in BCG non-responders. This complex connections between PD-L1 expression, immune cell infiltration, and treatment response reveal the multifaceted mechanisms underlying adaptive immune resistance and provide grounds for the potential for personalized therapeutic interventions targeting these pathways in NMIBC.

### 3.4. Critical Issues in PD-L1 Assessment 

Technical issues might also negatively influence assessment of PD-L1 status by immunohistochemical staining, including time length and type of tissue fixation, section preparation, and the quantity of tissue used (whole sections or tissue microarrays), resulting in poor reproducibility [74]. 

All type of tissue samples available in pathology routine have been used for PD-L1 IHC testing in different trials, including transurethral resection bladder (TURB), cystectomy, lymph node (LN), or visceral metastasis both pre- and post- (neo-)adjuvant chemotherapy [75], although how such variability can affect clinical findings has rarely been investigated. Generally, it is recommended to assess PD-L1 status on the most recent tumor sample collected during the disease course due to intratumoral heterogeneity. Eich et al. observed consistent differences in PD-L1 status assessing sequential TURBT specimens of NMIBC patients, with biopsies from the same patient showing variable to absent expression, irrespective of BCG treatment [48]. The PD-L1 intratumoral heterogeneity may be a major issue when evaluating a multifocal disease such as CIS, necessitating the sampling of more affected areas from the same patient rather than only one [61]. Tissue microarray (TMA) has been used in a few studies [51,60] to optimize the analysis of a number of tissue specimens by creating one tumor block which includes multiple cores of a few millimeters each, belonging to specimens from different patients. In order to overcome the issue of intratumoral heterogeneity, tissue microarray TMA can be applied only punching more cores (three or more) from the same tumor block [76]. 

Further technical issues affecting reproducibility of PD-L1 IHC analysis of TC are non-specific background staining and/or granular staining on crushed or necrotic tissue, stromal cells, and alveolar macrophages, as well as in case of a heavily keratinized or inflamed tumor. A reliable determination if total TC area may be hindered by the presence of a huge amount of necrosis, inflammation, or fibrosis at the edge of the tumor [77]. 

Different PD-L1 antibody clones are commercially available, each of them requiring specific technical platforms and scoring systems, prompting the need to assess the reliability and reproducibility of these tests and explore the analytical correlation among them [78]. Recently, the authors of an international comparison of PD-L1 diagnostic assays in a few cancers (the Ring Study) have reported interobserver comparison rates as high as k = 0.68–0.91 in UC for TC-PD-L1 (SP263) staining at 25% cut-off, in keeping with a previous clinical study [79]; conversely, low concordance (k= −0.04 to 0.76) was observed regarding the IC scoring [77]. Currently, atezolizumab is enlisted among the recommended regimens in first-line systemic therapy for cisplatin ineligible patients with locally advanced or metastatic BC [80]. Since the SP142 assay is the companion diagnostic for atezolizumab, patients’ tumor samples must be screened for PD-L1 (SP142) staining in ≥5% tumor-infiltrating ICs only across the tumor area. Since the tumor microenvironment hosting ICs is more easily adjustable across samples from the same patients as compared to genomically aberrant TCs, the SP263 assay may provide more stable results in that it takes into account TC-PD-L1 expression [81]. Moderate to substantial agreement, ranging from κ = 0.43 to k = 0.66, has been observed in studies comparing the SP142 and SP263 assays in whole-tissue sections and TMA of BC [81,82,83]. A fair agreement of staining interpretation was reported in a study on assessing PD-L1 expression in a cohort of CIS patients among 22C3, SP142, and SP263 in TC or IC [61]. According to several inter-assay agreement studies, concordance rates were higher between 22C3 and SP263 assay, as compared to those for SP142 [78,82,84,85]. While 22C3 and SP263 have been developed and validated for different platforms and identify epitope binding variance (extracellular domain and cytoplasmic domain, respectively), they both take TC and IC staining into account. On the other hand, the lower concordance levels between SP263 and SP142, which shares the same platform and epitope, highlight the major impact of different scoring system in affecting the reliable assessment of PD-L1 status [86]. As a result, altering assay protocols and/or quantification and interpretation methods might result in improving agreement among antibody clones [86]. Though used by a few studies, the E1L3N assay is a laboratory-developed antibody with unvalidated scoring system, thus accounting for the very low concordance with the above-mentioned commercially available clones, such as SP142 [87]. 

Pitfalls and issues of the IHC method can be overcome by measuring the PD-L1 mRNA level by quantitative reverse transcriptase-polymerase chain reaction (RT-PCR). Breyer et al. assessed the PD-L1 mRNA expression in a cohort of patients with T1 NMIBC tumors, treated with BCG and mitomycin C, reporting an association between high PD-L1 mRNA expression and significantly improved recurrence-free survival (RFS), progression-free survival (PFS), and cancer-specific survival (CSS) [70]. Furthermore, 5-year PFS was longer in patients treated with mitomycin C than with BCG (100% vs. 80%, respectively; *p* = 0.0788) [70]. In a later study from the same group, high-PD-L1 mRNA was an independent predictor of longer DSS and RFS by multivariate Cox’s regression analysis, regardless of their molecular subtype [88]. The implementation of PD-L1 assessment by RT-PCR in routine practice is hampered by many drawbacks in that this technique is money- and time-consuming and not as widespread as IHC. Furthermore, only IHC allows the identification of the localization of the molecule in TCs or ICs within the tissue by visualizing the antigen–antibody binding and quantifying its extent in each cell group.

In conclusion, the assessment of PD-L1 status in BC via IHC staining poses technical challenges such as tissue fixation, section preparation, and sample heterogeneity. Despite efforts to standardize protocols, variability in PD-L1 expression assessment persists, impacting treatment decisions and clinical outcomes. Inter-assay agreement studies have revealed discrepancies among PD-L1 antibody clones, highlighting the need for standardized scoring systems and assay protocols to ensure reliable and reproducible results. While RT-PCR offers an alternative method for PD-L1 assessment, its limited accessibility and inability to visualize PD-L1 localization within tissue highlight the ongoing need for refining immunohistochemical techniques for accurate PD-L1 evaluation in BC.

## 4. Conclusions

This comprehensive review elucidates the evolving landscape of PD-L1 as a prognostic biomarker in NMIBC, with a focus on Bacillus Calmette-Guérin (BCG) immunotherapy and on the interaction between PD-L1 and tumor microenvironment in this setting. The identification of PD-L1 as a potential prognostic factor not only suggests its use, alone or in combination, to refine the risk stratification of NMIBC patients, but also provides grounds for tailored therapeutic approaches, including immunotherapy with ICI, especially for patients showing resistance to conventional treatments. However, challenges such as intratumoral heterogeneity, technical issues in PD-L1 assessment, and discrepancies in study outcomes necessitate further efforts to establish standardized protocols. This review emphasizes the critical need for continued research to shine light on the prognostic role of PD-L1, offering insights that contribute to the ongoing debate on personalized treatment strategies for NMIBC patients.

## Figures and Tables

**Table 1:** Prognostic potential of PD-L1 in NMIBC as assessed by IHC, according to selected studies.

						Prevalence		Significant Correlation		Independent Predictor	
Study	N# Cases	Stage	Grade	Antibody Clone	Scoring System/Cut-off	TC	IC	TC	IC	TC	IC
[46]	55	Tis, Ta, T1	LG, HG	28-8	<1%—low; 1–5%—moderate; >5%—high expression	low 84%, moderate 10%, high 6%	low 34%, moderate 34%, high 32%	time to cystectomy, many recurrences	age, depth of bladder wall invasion	shorter RFS (high and moderate vs. low expression)	
[47]	140	Ta, T1	HG	SP142, SP263, 28-8, E1L3N	≥5% IC (SP142), ≥25% TC-IC (SP263), ROC curve (E1L3N)				recurrence/progression (E1L3N), pT1 stage (E1L3N, SP263)	pT1 stage (all antibodies)	shorter DFS (E1L3N)
[48]	61	CIS, Ta	LG, HG	E1L3N	1%, 5%			LG, lower recurrence risk	higher stage		
[50]	22	T1	HG	SP142	>1%	9%	73%	low peripheral blood lymphocytes			
[51]	140	T1	HG	405.9A11	5%(TC)	4%	34.3%	muscle invasion			
[52]	240	CIS, T1	LG, HG	SP263	≥25% TC-IC			shorter RFS in luminal tumors after no or BCG treatment			
[53]	59	Ta, T1	LG, HG	E1L3N	TCS, ICS, CS	18.6%	-	T1			
[58]	186	CIS, Ta, T1		E1L3N	>1%, >5%	18.8%		32.3%		immune infiltrate density, increased PD-L1 after BCG	
[59]	117	CIS, Ta, T1	HG	SP142	>1%	46.2%	refractory recurrence (inverse association)				
[60]	63	CIS, Ta, T1	HG	22C3, SP142	>5%, CPS			non responsiveness to BCG			
[61]	60	CIS		22C3, SP142, SP263	TPS, IC	16.7%	38.3%	failure to BCG therapy/recurrence (22C3)			
[62]	22	Tis, Ta, T1	G2, G3	E1L3N	IS, PS	9%	50%	BCG treatment			

BCG: Bacillus Calmette-Guérin; CIS: carcinoma in situ; CPS: combined positive score; CS: combined scoring; DFS: disease-free survival; HG: high grade; IC: immune cells; ICS: immune cell scoring; IS: intensity score; LG: low grade; PS: proportion score; RFS: recurrence-free survival; TC: tumor cells; TCS: tumor cell scoring; TPS: tumor proportion score.

## Data Availability

Not applicable.

## Supplementary Materials

The following supporting information can be downloaded at: https://www.mdpi.com/article/10.3390/jcm13082182/s1.

## Abbreviations

BC	Bladder cancer
BCG	Bacillus Calmette-Guérin
CIS	Carcinoma in situ
CSS	Cancer-specific survival
DFS	Disease-free survival
EMT	Epithelial-mesenchymal transition
HER2	Human Epidermal Growth Factor Receptor 2
HR-NMIBC	High-risk non-muscle-invasive bladder cancer
IHC	Immunohistochemistry
ICI	Immune checkpoint inhibitor
IC	Immune cells
IL-6	Interleukin 6
IFN-γ	Interferon γ
IFN-α	Interferon α
JAK	Janus kinases
LN	Lymph node
MeSH	Medical Subject Headings
MIBC	Muscle-invasive bladder cancer
NMIBC	Non-muscle-invasive bladder cancer
NLR	Neutrophil-to-lymphocyte ratio
OS	Overall survival
PD-L1	Programmed cell death ligand 1
PI3K	Phosphatidylinositol 3-kinases
PFS	Progression-free survival
re-TURB	re-transurethral resection of the bladder
RT-PCR	Reverse transcriptase-polymerase chain reaction
RFS	Recurrence-free survival
STAT3	Signal transducer and activator of transcription 3
STAT	Signal transducers and activators of transcription
TC	Tumor cells
TMA	Tissue microarray
TUR	Transurethral resection
TURB	Transurethral resection of tumor bladder

## References

[1] IARC-WHO Global Cancer Observatory. https://gco.iarc.fr.

[2] Netto G.J., Amin M.B., Berney D.M., Comperat E.M., Gill A.J., Hartmann A., Menon S., Raspollini M.R., Rubin M.A., Srigley J.R. (2022). The 2022 World Health Organization classification of tumors of the urinary system and male genital organs-part B: Prostate and urinary tract tumors. Eur. Urol..

[3] Compérat E., Larré S., Roupret M., Neuzillet Y., Pignot G., Quintens H., Houéde N., Roy C., Durand X., Varinot J. (2015). Clinicopathological characteristics of urothelial bladder cancer in patients less than 40 years old. Virchows Arch..

[4] Babjuk M., Burger M., Capoun O., Cohen D., Compérat E.M., Dominguez Escrig J.L., Gontero P., Liedberg F., Masson-Lecomte A., Mostafid A.H. (2022). European Association of Urology Guidelines on Non-muscle-invasive Bladder Cancer (Ta, T1, and Carcinoma in Situ). Eur. Urol..

[5] Calò B., Chirico M., Fortunato F., Sanguedolce F., Carvalho-Dias E., Autorino R., Carrieri G., Cormio L. (2019). Is Repeat Transurethral Resection Always Needed in High-Grade T1 Bladder Cancer?. Front. Oncol..

[6] Larsen E.S., Nordholm A.C., Lillebaek T., Holden I.K., Johansen I.S. (2019). The epidemiology of bacille Calmette-Guérin infections after bladder instillation from 2002 through 2017: A nationwide retrospective cohort study. BJU Int..

[7] Sanguedolce F., Russo D., Mancini V., Selvaggio O., Calo B., Carrieri G., Cormio L. (2019). Prognostic and therapeutic role of HER2 expression in micropapillary carcinoma of the bladder. Mol. Clin. Oncol..

[8] Sanguedolce F., Cormio A., Massenio P., Pedicillo M.C., Cagiano S., Fortunato F., Calò B., Di Fino G., Carrieri G., Bufo P. (2018). Altered expression of HER-2 and the mismatch repair genes MLH1 and MSH2 predicts the outcome of T1 high-grade bladder cancer. J. Cancer Res. Clin. Oncol..

[9] Keir M.E., Butte M.J., Freeman G.J., Sharpe A.H. (2008). PD-1 and its ligands in tolerance and immunity. Annu. Rev. Immunol..

[10] Afreen S., Dermime S. (2014). The immunoinhibitory B7-H1 molecule as a potential target in cancer: Killing many birds with one stone. Hematol. Oncol. Stem Cell Ther..

[11] Zou W., Wolchok J.D., Chen L. (2016). PD-L1 (B7-H1) and PD-1 pathway blockade for cancer therapy: Mechanisms, response biomarkers, and combinations. Sci. Transl. Med..

[12] Riley J.L. (2009). PD-1 signaling in primary T cells. Immunol. Rev..

[13] Dunn G.P., Old L.J., Schreiber R.D. (2004). The immunobiology of cancer immunosurveillance and immunoediting. Immunity.

[14] Kim J.H., Park H.E., Cho N.Y., Lee H.S., Kang G.H. (2016). Characterisation of PD-L1-positive subsets of microsatellite-unstable colorectal cancers. Br. J. Cancer.

[15] Zhang W.T., Zhang J.F., Zhang Z.W., Guo Y.D., Wu Y., Wang R., Wang L., Mao S., Yao X. (2019). Overexpression of indoleamine 2,3-dioxygenase 1 promotes epithelial–mesenchymal transition by activation of the IL-6/STAT3/PD-L1 pathway in bladder cancer. Transl. Oncol..

[16] Parsa A.T., Waldron J.S., Panner A., Crane C.A., Parney I.F., Barry J.J., Cachola K.E., Murray J.C., Tihan T., Jensen M.C. (2007). Loss of tumor suppressor PTEN function increases B7-H1 expression and immunoresistance in glioma. Nat. Med..

[17] Van Allen E.M., Golay H.G., Liu Y., Koyama S., Wong K., Taylor-Weiner A., Giannakis M., Harden M., Rojas-Rudilla V., Chevalier A. (2015). Long-term benefit of PD-L1 blockade in lung cancer associated with JAK3 activation. Cancer Immunol. Res..

[18] Powles T., Eder J.P., Fine G.D., Braiteh F.S., Loriot Y., Cruz C., Bellmunt J., Burris H.A., Petrylak D.P., Teng S.L. (2014). MPDL3280A (anti-PD-L1) treatment leads to clinical activity in metastatic bladder cancer. Nature.

[19] Parvez A., Choudhary F., Mudgal P., Khan R., Qureshi K.A., Farooqi H., Aspatwar A. (2023). PD-1 and PD-L1: Architects of immune symphony and immunotherapy breakthroughs in cancer treatment. Front. Immunol..

[20] Nadal R., Valderrama B.P., Bellmunt J. (2024). Progress in systemic therapy for advanced-stage urothelial carcinoma. Nat. Rev. Clin. Oncol..

[21] de Jong F.C., Rutten V.C., Zuiverloon T.C.M., Theodorescu D. (2021). Improving Anti-PD-1/PD-L1 Therapy for Localized Bladder Cancer. Int. J. Mol. Sci..

[22] Huang S., Huang Y., Li C., Liang Y., Huang M., Luo R., Liang W. (2024). Efficacy and safety of neoadjuvant PD-1 inhibitors or PD-L1 inhibitors for muscle invasive bladder cancer: A systematic review and meta-analysis. Front. Immunol..

[23] Song M., Chen D., Lu B., Wang C., Zhang J., Huang L., Wang X., Timmons C.L., Hu J., Liu B. (2013). PTEN loss increases PD-L1 protein expression and affects the correlation between PD-L1 expression and clinical parameters in colorectal cancer. PLoS ONE.

[24] Choueiri T.K., Fay A.P., Gray K.P., Callea M., Ho T.H., Albiges L., Bellmunt J., Song J., Carvo I., Lampron M. (2014). PD-L1 expression in nonclear-cell renal cell carcinoma. Ann. Oncol..

[25] Berghoff A.S., Kiesel B., Widhalm G., Rajky O., Ricken G., Wöhrer A., Dieckmann K., Filipits M., Brandstetter A., Weller M. (2015). Programmed death ligand 1 expression and tumor-infiltrating lymphocytes in glioblastoma. Neuro Oncol..

[26] Riobello C., Vivanco B., Reda S., López-Hernández A., García-Inclán C., Potes-Ares S., Cabal V.N., López F., Llorente J.L., Hermsen M.A. (2018). Programmed death ligand-1 expression as immunotherapeutic target in sinonasal cancer. Head Neck.

[27] Cha Y.J., Kim H.R., Lee C.Y., Cho B.C., Shim H.S. (2016). Clinicopathological and prognostic significance of programmed cell death ligand-1 expression in lung adenocarcinoma and its relationship with p53 status. Lung Cancer.

[28] Jiang D., Xu Y.Y., Li F., Xu B., Zhang X.G. (2014). The role of B7-H1 in gastric carcinoma: Clinical significance and related mechanism. Med. Oncol..

[29] Zhao L.W., Li C., Zhang R.L., Xue H.G., Zhang F.X., Zhang F., Gai X.D. (2014). B7-H1 and B7-H4 expression in colorectal carcinoma: Correlation with tumor FOXP3(+) regulatory T-cell infiltration. Acta Histochem..

[30] Gao H.L., Liu L., Qi Z.H., Xu H.X., Wang W.Q., Wu C.T., Zhang S.R., Xu J.Z., Ni Q.X., Yu X.J. (2018). The clinicopathological and prognostic significance of PD-L1 expression in pancreatic cancer: A meta-analysis. Hepatobiliary Pancreat. Dis. Int..

[31] Inman B.A., Sebo T.J., Frigola X., Dong H., Bergstralh E.J., Frank I., Fradet Y., Lacombe L., Kwon E.D. (2007). PD-L1 (B7-H1) expression by urothelial carcinoma of the bladder and BCG-induced granulomata: Associations with localized stage progression. Cancer.

[32] Boorjian S.A., Frank I., Lohse C.M., Kuntz S.M., Leibovich B.C., Kwon E.D., Frank I. (2008). T cell coregulatory molecule expression in urothelial cell carcinoma: A potential target for therapy. J. Urol..

[33] Xylinas E., Robinson B.D., Kluth L.A., Volkmer B.G., Hautmann R., Küfer R., Zerbib M., Kwon E., Thompson R.H., Boorjian S.A. (2014). Association of T-cell co-regulatory protein expression with clinical outcomes following radical cystectomy for urothelial carcinoma of the bladder. Eur. J. Surg. Oncol..

[34] Faraj S.F., Munari E., Guner G., Taube J., Anders R., Hicks J., Meeker A., Schoenberg M., Bivalacqua T., Drake C. (2015). Assessment of tumoral PD-L1 expression and intratumoral CD8+ T cells in urothelial carcinoma. Urology.

[35] Nakanishi J., Wada Y., Matsumoto K., Azuma M., Kikuchi K., Ueda S. (2007). Overexpression of B7-H1 (PD-L1) significantly associates with tumor grade and postoperative prognosis in human urothelial cancers. Cancer Immunol. Immunol..

[36] Boorjian S.A., Sheinin Y., Crispen P.L., Farmer S.A., Lohse C.M., Kuntz S.M., Leibovich B.C., Kwon E.D., Frank I. (2008). T-cell coregulatory molecule expression in urothelial cell carcinoma: Clinicopathologic correlations and association with survival. Clin. Cancer Res..

[37] Pichler R., Heidegger I., Fritz J., Danzl M., Sprung S., Zelger B., Brunner A., Pircher A. (2017). PD-L1 expression in bladder cancer and metastasis and its influence on oncologic outcome after cystectomy. Oncotarget..

[38] Sharma P., Retz M., Siefker-Radtke A., Baron A., Necchi A., Bedke J., Plimack E.R., Vaena D., Grimm M.O., Bracarda S. (2017). Nivolumab in metastatic urothelial carcinoma after platinum therapy (Check-Mate 275): A multicentre, single-arm, phase 2 trial. Lancet Oncol..

[39] Erlmeier F., Seitz A.K., Hatzichristodoulou G., Stecher L., Retz M., Gschwend J.E., Weichert W., Kübler H.R., Horn T. (2016). The role of PD-L1 expression and intratumoral lymphocytes in response to perioperative chemotherapy for urothelial carcinoma. Bladder Cancer.

[40] Bellmunt J., Mullane S.A., Werner L., Fay A.P., Callea M., Leow J.J., Taplin M.E., Choueiri T.K., Hodi F.S., Freeman G.J. (2015). Association of PD-L1 expression on tumor-infiltrating mononuclear cells and overall survival in patients with urothelial carcinoma. Ann. Oncol..

[41] Wen Y., Chen Y., Duan X., Zhu W., Cai C., Deng T., Zeng G. (2019). The clinicopathological and prognostic value of PD-L1 in urothelial carcinoma: A meta-analysis. Clin. Exp. Med..

[42] Ding X., Chen Q., Yang Z., Li J., Zhan H., Lu N., Chen M., Yang Y., Wang J., Yang D. (2019). Clinicopathological and prognostic value of PD-L1 in urothelial carcinoma: A meta-analysis. Cancer Manag. Res..

[43] Zhu L., Sun J., Wang L., Li Z., Wang L., Li Z. (2019). Prognostic and Clinicopathological Significance of PD-L1 in Patients With Bladder Cancer: A Meta-Analysis. Front. Pharmacol..

[44] Liu H., Ye T., Yang X., Lv P., Wu X., Zhou H., Lu H., Tang K., Ye Z. (2020). Predictive and Prognostic Role of PD-L1 in Urothelial Carcinoma Patients with Anti-PD-1/PD-L1 Therapy: A Systematic Review and Meta-Analysis. Dis. Markers.

[45] Zhang J., Song L., Zhu H., Liu Q., Wang D. (2022). Prognostic value of programmed cell death ligand-1 expression in patients with bladder urothelial carcinoma undergoing radical cystectomy: A meta-analysis. Front. Immunol..

[46] Semeniuk-Wojtaś A., Modzelewska M., Poddębniak-Strama K., Kołaczyńska S., Lubas A., Górnicka B., Jakieła A., Stec R. (2023). CD4, CD20 and PD-L1 as Markers of Recurrence in Non-Muscle-Invasive Bladder Cancer. Cancers.

[47] Roumiguié M., Compérat E., Chaltiel L., Nouhaud F.X., Verhoest G., Masson-Lecomte A., Colin P., Audenet F., Houédé N., Larré S. (2021). PD-L1 expression and pattern of immune cells in pre-treatment specimens are associated with disease-free survival for HR-NMIBC undergoing BCG treatment. World J. Urol..

[48] Eich M.L., Chaux A., Guner G., Taheri D., Mendoza Rodriguez M.A., Rodriguez Peña M.D.C., Baras A.S., Hahn N.M., Drake C., Sharma R. (2019). Tumor immune microenvironment in non-muscle-invasive urothelial carcinoma of the bladder. Hum. Pathol..

[49] Eckstein M., Wirtz R.M., Pfannstil C., Wach S., Stoehr R., Breyer J., Erlmeier F., Günes C., Nitschke K., Weichert W. (2018). A multicenter round robin test of PD-L1 expression assessment in urothelial bladder cancer by immunohistochemistry and RT-qPCR with emphasis on prognosis prediction after radical cystectomy. Oncotarget.

[50] Martınez R., Tapia G., De Muga S., Hernandez A., Cao M.G., Teixido C., Urrea V., García E., Pedreño-López S., Ibarz L. (2019). Combined assessment of peritumoral Th1/Th2 polarization peripheral immunity as a new biomarker in the prediction of BCG response in patients with high-risk, NMIBC. Oncoimmunology.

[51] Wankowicz S.A.M., Werner L., Orsola A., Novak J., Bowden M., Choueiri T.K., de Torres I., Morote J., Freeman G.J., Signoretti S. (2017). Differential Expression of PD-L1 in High Grade T1 vs Muscle Invasive Bladder Carcinoma and its Prognostic Implications. J. Urol..

[52] Blinova E., Enikeev D., Roshchin D., Samyshina E., Deryabina O., Tertychnyy A., Blinov D., Kogan E., Dudina M., Barakat H. (2020). Relapse-Free Survival and PD-L1 Expression in First High- and Low-Grade Relapsed Luminal, Basal and Double-Negative P53-Mutant Non-Muscular Invasive Bladder Cancer Depending on Previous Chemo- and Immunotherapy. Cancers.

[53] Civriz A.H., Teke K., Akdas E.M., Dillioglugil O., Vural C., Yaprak Bayrak B. (2023). The prognostic value of expressions of STAT3, PD-L1, and PD-L2 in Ta/T1 urothelial carcinoma before and after BCG treatment. Urol. Oncol..

[54] van der Horst G., Bos L., van der Pluijm G. (2012). Epithelial plasticity, cancer stem cells, and the tumor-supportive stroma in bladder carcinoma. Mol. Cancer Res..

[55] Huang Y., Zhang S.D., McCrudden C., Chan K.W., Lin Y., Kwok H.F. (2015). The prognostic significance of PD-L1 in bladder cancer. Oncol. Rep..

[56] Powles T., Duran I., van der Heijden M.S., Loriot Y., Vogelzang N.J., De Giorgi U., Oudard S., Retz M.M., Castellano D., Bamias A. (2018). Atezolizumab versus chemotherapy in patients with platinum-treated locally advanced or metastatic urothelial carcinoma (IMvigor211): A multicentre, open-label, phase 3 randomised controlled trial. Lancet.

[57] Powles T., O’Donnell P.H., Massard C., Arkenau H.T., Friedlander T.W., Hoimes C.J., Lee J.L., Ong M., Sridhar S.S., Vogelzang N.J. (2017). Efficacy and safety of durvalumab in locally advanced or metastatic urothelial carcinoma: Updated results from a phase 1/2 OPEN-LABEL STUDY. JAMA Oncol..

[58] Delcourt C., Gemival P., Nouhaud F.X., Gobet F., Gillibert A., Ferlicot S., Sabourin J.C., Irani J., Pfister C. (2020). Clinical interest of PD-L1 immuno-histochemistry expression as a predictive factor of Bacillus Calmette Guerin (BCG) efficacy in refractory high-risk non-muscle-invasive bladder cancer (NMIBC). World J. Urol..

[59] Aydin A.M., Baydar D.E., Hazir B., Babaoglu B., Bilen C.Y. (2020). Prognostic significance of pre- and post-treatment PD-L1 expression in patients with primary high-grade non-muscle-invasive bladder cancer treated with BCG immunotherapy. World J. Urol..

[60] Kates M., Matoso A., Choi W., Baras A.S., Daniels M.J., Lombardo K., Brant A., Mikkilineni N., McConkey D.J., Kamat A.M. (2020). Adaptive immune resistance to intravesical BCG in non-muscle invasive bladder cancer: Implications for prospective BCG unresponsive trials. Clin. Cancer Res..

[61] Pierconti F., Raspollini M.R., Martini M., Larocca L.M., Bassi P.F., Bientinesi R., Baroni G., Minervini A., Petracco G., Pini G.M. (2020). PD-L1 expression in bladder primary in situ urothelial carcinoma: Evaluation in BCG-unresponsive patients and BCG responders. Virchows Arch..

[62] Hashizume A., Umemoto S., Yokose T., Nakamura Y., Yoshihara M., Shoji K., Wada S., Miyagi Y., Kishida T., Sasada T. (2018). Enhanced expression of PD-L1 in non-muscle-invasive bladder cancer after treatment with Bacillus Calmette-Guerin. Oncotarget.

[63] Kamat A.M., Shore N., Hahn N., Alanee S., Nishiyama H., Shariat S., Nam K., Kapadia E., Frenkl T., Steimberg G. (2020). KEYNOTE-676: Phase III study of BCG and pembrolizumab for persistent/recurrent high-risk NMIBC. Future Oncol..

[64] A Phase III Randomized, Open-Label, Multi-Center, Global Study of Durvalumab and Bacillus Calmette-Guerin (BCG) Administered as Combination Therapy Versus BCG Alone in High-Risk, BCG Naïve Non-Muscle Invasive Bladder Cancer Patients (POTOMAC) EudraCT Number: L2017-002979-26. https://www.clinicaltrialsregister.eu/ctr-search/trial/2017-002979-26/SK.

[65] An Open Label, Randomized, Phase III Trial, Evaluating Efficacy of Atezolizumab in Addition to One Year BCG (Bacillus Calmette-Guérin) Bladder Instillation in BCG-Naive Patients with High-Risk Non-Muscle Invasive Bladder Cancer. Eudract Number: 2017-004512-19. https://www.clinicaltrialsregister.eu/ctr-search/trial/2017-004512-19/ES.

[66] Nowak Ł., Krajewski W., Poterek A., Śliwa A., Zdrojowy R. (2020). The prognostic value of programmed cell death protein ligand 1 in patients with non-muscle-invasive bladder cancer treated with bacille Calmette-Guérin immunotherapy: Current status. Arab J. Urol..

[67] Topalian S.L., Taube J.M., Anders R.A., Pardoll D.M. (2016). Mechanism-driven biomarkers to guide immune checkpoint blockade in cancer therapy. Nat. Rev. Cancer.

[68] Miller E.J., Galsky M.D. (2023). Precision Medicine in Urothelial Carcinoma: Current Markers to Guide Treatment and Promising Future Directions. Curr. Treat. Options Oncol..

[69] Goutas D., Palamaris K., Stofas A., Politakis N., Despotidi A., Giannopoulou I., Goutas N., Vlachodimitropoulos D., Kavantzas N., Lazaris A.C. (2022). Immunohistochemical Study of Bladder Cancer Molecular Subtypes and Their Association with PD-L1 Expression. Cancers.

[70] Breyer J., Wirtz R.M., Otto W., Erben P., Worst T.S., Stoehr R., Eckstein M., Denzinger S., Burger M., Hartmann A. (2018). High PDL1 mRNA expression predicts better survival of stage pT1 non-muscle-invasive bladder cancer (NMIBC) patients. Cancer Immunol. Immunother..

[71] Agarwal A., Agrawal U., Verma S., Mohanty N.K., Saxena S. (2010). Serum Th1 and Th2 cytokine balance in patients of superficial transitional cell carcinoma of bladder pre- and post-intravesical combination immunotherapy. Immunopharmacol. Immunotoxicol..

[72] Pichler R., Gruenbacher G., Culig Z., Brunner A., Fuchs D., Fritz J., Gander H., Rahm A., Thurnher M. (2017). Intratumoral Th2 predisposition combines with an increased Th1 functional phenotype in clinical response to intravesical BCG in bladder cancer. Cancer Immunol. Immunother..

[73] Chevalier M.F., Schneider A.K., Cesson V., Dartiguenave F., Lucca I., Jichlinski P., Nardelli-Haefliger D., Derré L. (2018). Conventional and PD-L1-expressing Regulatory T Cells are Enriched During BCG Therapy and may Limit its Efficacy. Eur. Urol..

[74] Paver E.C., Cooper W.A., Colebatch A.J., Ferguson P.M., Hill S.K., Lum T., Shin J.S., O’Toole S., Anderson L., Scolyer R.A. (2021). Programmed death ligand-1 (PD-L1) as a predictive marker for immunotherapy in solid tumours: A guide to immunohistochemistry implementation and interpretation. Pathology.

[75] Rouanne M., Radulescu C., Adam J., Allory Y. (2021). PD-L1 testing in urothelial bladder cancer: Essentials of clinical practice. World J. Urol..

[76] Rouanne M., Lebret T., Radulescu C., Adam J. (2018). Re: Differential Expression of PD-L1 in High Grade T1 vs. Muscle Invasive Bladder Carcinoma and its Prognostic Implications: Wankowicz, S.A.M.; Werner, L.; Orsola, A.; Novak, J.; Bowden, M.; Choueiri, T.K.; de Torres, I.; Morote, J.; Freeman, G.J.; Signoretti, S.; Bellmunt, J. *J. Urol.*
**2017**, *198*, 817–823. J. Urol..

[77] Yu S.L., Hsiao Y.J., Cooper W.A., Choi Y.L., Avilés-Salas A., Chou T.Y., Coudry R., Raskin G.A., Fox S.B., Huang C.C. (2023). The Ring Study: An international comparison of PD-L1 diagnostic assays and their interpretation in non-small cell lung cancer, head and neck squamous cell cancer and urothelial cancer. Pathology.

[78] Tretiakova M., Fulton R., Kocherginsky M., Long T., Ussakli C., Antic T., Gown A. (2018). Concordance study of PD-L1 expression in primary and metastatic bladder carcinomas: Comparison of four commonly used antibodies and RNA expression. Mod. Pathol..

[79] Zajac M., Boothman A.M., Ben Y., Gupta A., Jin X., Mistry A., Sabalos C., Nielsen A., Manriquez G., Barker C. (2019). Analytical validation and clinical utility of an immunohistochemical programmed death ligand-1 diagnostic assay and combined tumor and immune cell scoring algorithm for durvalumab in urothelial carcinoma. Arch. Pathol. Lab. Med..

[80] NCCN Clinical Practice Guidelines in Oncology (NCCN Guidelines®). Bladder Cancer. Version 1.2024—30 January 2024. https://www.nccn.org/professionals/physician_gls/pdf/bladder.pdf.

[81] de Jong J.J., Stoop H., Boormans J.L., van Leenders G.J.L.H. (2021). PD-L1 expression in urothelial bladder cancer varies more among specimen types than between companion assays. Virchows Arch..

[82] Rijnders M., van der Veldt A.A.M., Zuiverloon T.C.M., Grünberg K., Thunnissen E., de Wit R., van Leenders G.J.L.H. (2019). PD-L1 anti-body comparison in urothelial carcinoma. Eur. Urol..

[83] Wang C., Hahn E., Slodkowska E., Eskander A., Enepekides D., Higgins K., Vesprini D., Liu S.K., Downes M.R., Xu B. (2018). Reproducibility of PD-L1 immunohistochemistry interpretation across various types of genitourinary and head/neck carcinomas, antibody clones, and tissue types. Hum. Pathol..

[84] Eckstein M., Erben P., Kriegmair M.C., Worst T.S., Weiß C.A., Wirtz R.M., Wach S., Stoehr R., Sikic D., Geppert C.I. (2019). Performance of the Food and Drug Administration/EMA-approved programmed cell death ligand-1 assays in urothelial carcinoma with emphasis on therapy stratification for first-line use of atezolizumab and pembrolizumab. Eur. J. Cancer.

[85] Hodgson A., Slodkowska E., Jungbluth A., Liu S.K., Vesprini D., Enepekides D., Higgins K., Katabi N., Xu B., Downes M.R. (2018). PD-L1 immunohistochemistry assay concordance in urothelial carcinoma of the bladder and hypopharyngeal squamous cell carcinoma. Am. J. Surg. Pathol..

[86] Lawson N.L., Dix C.I., Scorer P.W., Stubbs C.J., Wong E., Hutchinson L., McCall E.J., Schimpl M., DeVries E., Walker J. (2019). Mapping the binding sites of antibodies utilized in programmed cell death ligand-1 predictive immunohistochemical assays for use with immuno-oncology therapies. Mod. Pathol..

[87] McLaughlin J., Han G., Schalper K.A., Carvajal-Hausdorf D., Pele-kanou V., Rehman J., Velcheti V., Herbst R., LoRusso P., Rimm D.L. (2016). Quantitative assessment of the heterogeneity of PD-L1 expression in non-small-cell lung cancer. JAMA Oncol..

[88] Kubon J., Sikic D., Eckstein M., Weyerer V., Stöhr R., Neumann A., Keck B., Wullich B., Hartmann A., Wirtz R.M. (2020). Analysis of CXCL9, PD1 and PD-L1 mRNA in Stage T1 Non-Muscle Invasive Bladder Cancer and Their Association with Prognosis. Cancers.

